# Supplementary material for: Muscle Damage in Dystrophic mdx Mice Is Influenced by the Activity of Ca2+-Activated KCa3.1 Channels
Source: Life (Basel). 2022 Apr 5;12(4):538. doi: 10.3390/life12040538 (PMC9025295; doi:10.3390/life12040538)
Supplement: Supplementary file 1 [file life-12-00538-s001.zip › life-1638842-supplementary.pdf]

life

MDPI

Type of the Paper (Article)

Supplementary Tables and Figures

Table S1: Primers used for RT-PCR.

Gene	Forward (F)	Reverse (R)
GAPDH	5′-TCGTCCCGTAGACAAAATGG-3′	5′-TTGAGGTCAATGAAGGGGTC- 3′
Kccn4	5′-GGCTGAAACACCGGAAGCTC-3′	5′-CAGCTCTGTCAGGGCATCCA-3′
Arg1	5′- CTCCAAGCCAAAGTCCTTAGAG-3′	5′- AGGAGCTGTCATTAGGGACATC-3′
Inos	5′-ACATCGACCCGTCCACAGTAT-3′	5′- CAGAGGGGTAGGCTTGTCTC-3
CD206	5′-CAAGGAAGGTTGGCATTTGT-3′	5′-CCTTTCAGTCCTTTGCAAGC-3′
Fibronectin	5′-GAGCCTTCACACATCACCAA-3′	5′-TAAGGTGGCCAGGAATGGTA-3′
Coll1a1	5′-TGGCAAGAATGGAGATGATG-3′	5′-CCATCCAAACCACTGAAGC-3′

Table S2: Macrophage and muscle characteristics in mice treated from 3 weeks of age.

	Vehicle (n=10)	TRAM-34 (n=10)	% change	Effect Size	P
$iba1^+/mm^2$ (Dia)	137 ± 36	156 ± 29	+14 %	0.6	0.24
$arginase^+/mm^2$ (Dia)	67 ± 15	105 ± 22	+57 %	2.0	0.0017
$arginase^+/iba1^+$ (Dia)	0.50± 0.03	0.67 ± 0.04	+34 %	4.6	0.0002
Collagen-covered surface (Dia)	13.2 ± 2.2 %	9.6 ± 1.9 %	-27 %	1.73	0.0017
Collagen-covered surface (TA)	9.9 ± 1.8	7.4 ± 1.8	-25 %	1.4	0.006
Fibers with central nuclei (Dia)	31 ± 7 %	21 ± 4 %	-32 %	1.35	0.01
Fibers with central nuclei (TA)	47 ± 7 %	35 ± 8 %	-26 %	1.67	0.012
minimal Feret's diameter (Dia)	22.6± 4.4	19.52 ± 0.77	-14 %	0.98	0.04
z (Dia)	402 ± 30	360 ± 37	-10 %	1.25	0.014

Macrophage reactivity, collagen deposition and fiber characteristics in diaphragm (Dia) or Tibialis Anterior (TA) of mdx mice treated between 3 and 8 weeks of age. *z*: variance coefficient of minimal Feret's diameter; % Change = 100 * ($mean_{TRAM-34}$ - $mean_{vehicle}$)/$mean_{vehicle}$; Effect size was calculated as Cohen's D; P was calculated using 2-tailed Mann-Whitney test

Table S3: Macrophage and muscle characteristics in mice treated from 5 weeks of age.

	Vehicle (n=6)	TRAM-34 (n=6)	% change	Effect Size	P
$iba1^+/mm^2$ (Dia)	226 ± 28	285 ± 49	+26 %	1.46	0.066
$arginase^+/mm^2$ (Dia)	94 ± 28	149 ± 34	+58 %	1.76	0.02
$arginase^+/iba1^+$ (Dia)	0.40 ± 0.06	0.52 ± 0.05	+30 %	2.06	0.013
Collagen-covered surface (Dia)	11.3 ± 1.4 %	6.7 ± 1.0 %	-41 %	3.83	0.0051
Collagen-covered surface (TA)	7.80 ± 0.74 %	5.5 ± 1.1 %	-30 %	2.47	0.0083
Fibres with central nuclei (Dia)	37 ± 11 %	30 ± 8 %	-19 %	0.68	0.4
Fibres with central nuclei (TA)	54 ± 9 %	48 ± 15 %	-11 %	0.5	0.4
minimal Feret's diameter (Dia)	23.5 ± 2.0	22.3 ± 1.5	-5 %	0.69	0.31
z (Dia)	424 ± 37	448 ± 24	+5.6 %	0.79	0.25

Macrophage reactivity, collagen deposition and fiber characteristics in diaphragm (Dia) or Tibialis Anterior (TA) of mdx mice treated between 5 and 9 weeks of age. *z*: variance coefficient of minimal Feret's diameter; % Change = 100 * ($mean_{TRAM-34}$ - $mean_{vehicle}$)/$mean_{vehicle}$; Effect size was calculated as Cohen's D; P was calculated using 2-tailed Mann-Whitney test

Table S4: Macrophage and muscle characteristics in mice treated from 15 weeks of age.

	Vehicle (n=6)	TRAM-34 (n=6)	% change	Effect Size	P
$iba1^+/mm^2$ (Dia)	396 ± 32	497 ± 42	+25%	2.7	0.00094
$arginase^+/mm^2$ (Dia)	258 ± 38	366 ± 30	+42%	3.1	0.0083
$arginase^+/iba1^+$ (Dia)	0.65 ± 0.04	0.74 ± 0.02	+14%	2.7	0.016
Collagen-covered surface (Dia)	15.2 ± 2.8	11.0 ± 1.5	-28 %	2.93	0.02
Collagen-covered surface (TA)	11.2 ± 1.4	8.62 ± 0.57	-23 %	2.47	0.01
Fibres with central nuclei (Dia)	79 ± 17 %	75 ± 16 %	- 5.0 %	0.18	0.74
Fibres with central nuclei (TA)	73 ± 4 %	70 ± 8 %	-4.1 %	0.33	0.58
minimal Feret's diameter(Dia)	20.1±0.85	20.4±0.57	1.5 %	0.38	0.52
z (Dia)	407 ± 27	385 ± 25	-5.4%	0.86	0.17

Macrophage reactivity, collagen deposition and fiber characteristics in diaphragm (Dia) or Tibialis Anterior (TA) of mdx mice treated between 15 and 19 weeks of age. *z*: variance coefficient of minimal Feret's diameter; % Change = 100 * ($mean_{TRAM-34}$ - $mean_{vehicle}$)/$mean_{vehicle}$; Effect size was calculated as Cohen's D; P was calculated using 2-tailed Mann-Whitney test

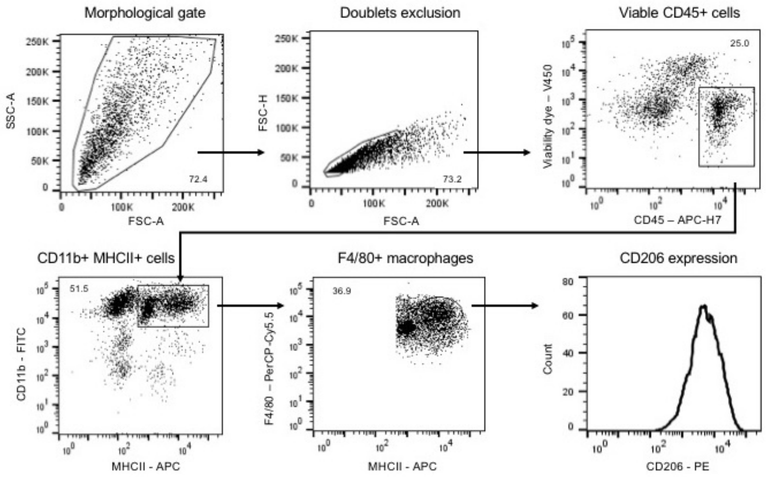

Figure S1: Gating strategy. Representative gating strategy used to identify infiltrating macrophages by flow cytometry in muscle samples from mdx mice. First, Debris were gated out in the FSC (forward scatter) vs SSC (side scatter) plot; then, singlets, live and CD45+ cells were considered; finally, macrophages were defined as CD11b+ MHC Class II+ F4/80+ cells; CD206 expression was evaluated by median fluorescence intensity. Numbers in the plots indicate the percentage of gated cells with respect to parent cells.

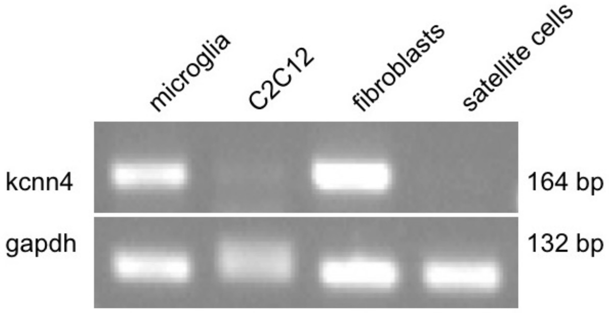

Figure S2: Expression of Kcnn4 gene. Representative image of PCR experiments showing expression ok kcnn4 gene in microglia (positive control) and cultured primary fibroblasts from mdx muscle, but not satellite cells purified from mdx muscles or myogenic C2C12 cell line.

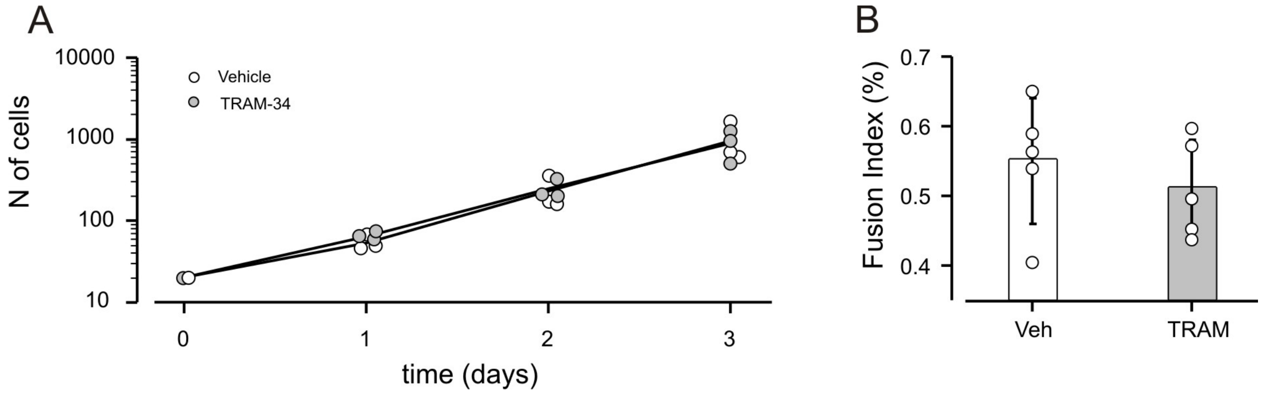

Figure S3: KCa3.1 channels play no role in proliferation and fusion of C2C12 myoblasts. (A) Number of cells counted in vehicle- or TRAM-34-treated dishes at the indicated times (3 experiments, each with 2 dishes/time point). Day 0: time of plating. (B) Fusion index of cells exposed to differentiative medium for 48 hours in the presence of vehicle- or TRAM-34, calculated according to the formula: $FI = 100 \frac{N\, nuclei\, in\, multinucleated\, cells}{N\, nuclei\, in\, the\, field}$, scoring six to ten fields on each of 5 Petri dishes (3 different platings) .
